# Chronic Inflammatory Gingival Enlargement: A Case Report

**DOI:** 10.7759/cureus.54296

**Published:** 2024-02-16

**Authors:** Aakanksha V Tiwari, Suwarna Dangore-Khasbage, Swapnil Mohod

**Affiliations:** 1 Oral Medicine and Radiology, Sharad Pawar Dental College and Hospital, Datta Meghe Institute of Higher Education & Research, Wardha, IND

**Keywords:** local factors, inflammation, gingiva, enlargement, calculus

## Abstract

Gingival enlargement can be referred to as an increased size of the gingival tissues. It might have originated because of inflammation, induced by certain drugs, linked to generalized illness, malignant, or pseudo enlargement, based on its etiology and pathogenesis. Enlargements may be widespread, papillary, or marginal, depending on the location. It affects the patient's masticatory, functional, aesthetic, and psychological health. Diagnosing the condition and its underlying cause through a detailed history is the mainstay for management. Diagnosis is based on a careful clinical examination in relation to the consistency, texture, and color of enlarged gingival tissues. Once diagnosed, the intervention relies on treating the causative factor involved in the condition. Early diagnosis and treatment with the elimination of the etiologic factor along with strict oral hygiene instructions and regular follow-up results in the restoration of aesthetics and function. This article presents a case report of a female patient aged 31 years who presented to the Oral Medicine and Radiology Department with chief complaints of swollen gums, bleeding gums while brushing for one month, and pain and loosening of teeth in the upper left back region of the jaw since 15 days. On thorough clinical examination, oral hygiene instructions were given along with antimicrobials and analgesic medications, and extensive scaling and sub-gingival curettage were done. On the follow-up visit after seven days, there was reduced inflammation due to the removal of local irritants like plaque and calculus and reduced gingival enlargement. The takeaway message from this case is that clinicians should be thoroughly acquainted with the normal and pathologic alterations of the gingival tissues and possible etiologic factors for it. Careful examination, prompt diagnosis, and treatment form the mainstay of management.

## Introduction

Increased gingival size is known as gingival expansion or overgrowth, and it is a common characteristic of diseases related to the gingival tissues. Accurately verifying the etiology proves essential for a timely intervention. Nonetheless, among the plethora of diseases that may be categorized based on causative factors and altered architecture, exact location and its spread, and/or grade of enlargement, the skills of clinicians are tested when reaching an accurate diagnosis. It might have originated because of inflammation, induced by certain drugs, linked to generalized illness, malignant, or fake, based on the etiology and pathogenesis. Enlargements may be widespread, papillary, or marginal, depending on the location [1].

It is a rare disorder that affects people's masticatory, functional, aesthetic, and psychological health. If the etiology of the condition is obvious, a clinical diagnosis of gingival enlargement may be made quickly. However, in order to govern the most suitable course of action, a doctor may be consulted in order to investigate the problem and rule out underlying illnesses, drug interactions, or physiological abnormalities. A precise diagnosis and, thus, a prognosis are difficult to establish when the etiology is unknown [2].

Involvement around three teeth or more in a particular or multiple locations of the oral cavity is termed to be "regional" enlargement (e.g., enlargement that is restricted to maxillary and mandibular anterior region caused due to mouth breathing). When the gingiva surrounding nearly every tooth is affected, it is termed as a "generalized" expansion (e.g., gingival overgrowth caused due to certain medications). A summary of different modalities and their clinical indices for gauging the spread of the condition was provided by Ingles et al. [3].

In this article, a case of a female patient aged 31 years is presented, who reported chief complaints of swelling of the gums, bleeding gums while brushing, and pain and loosening of teeth in the upper left back region of the jaw.

## Case presentation

Description of the case

A female aged 31 years presented to the Oral Medicine and Radiology Department, with complaints of swollen gums and bleeding gums while brushing for one month and pain and loosening of teeth in the upper left back region of the jaw for 15 days. There was no relevant systemic history of hypertension, diabetes mellitus, epilepsy, thyroid, or asthma and no relevant family history. The patient underwent oral prophylaxis one year ago. She was not addicted to any adverse habits and did not give a history of allergic reactions to any medication to date. She mentioned that she has been using Agrow Dant Manjan for brushing her teeth for the past two to three years. On extra-oral examination, her face was grossly symmetrical, temporomandibular joint jaw movements were bilaterally synchronous, and regional lymphadenopathy was absent.

**Figure 1:**
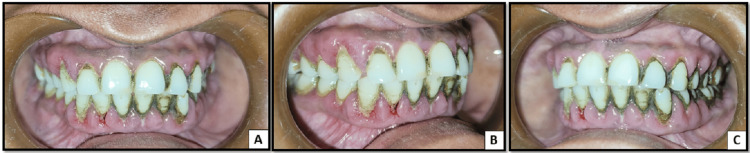
A: Intraoral picture depicting gingival inflammation and enlargement in the anterior region. B: Intraoral picture depicting gingival inflammation and enlargement in the posterior region on the right side. C: Intraoral picture depicting gingival inflammation and enlargement in the posterior region on the left side.

**Figure 2:**
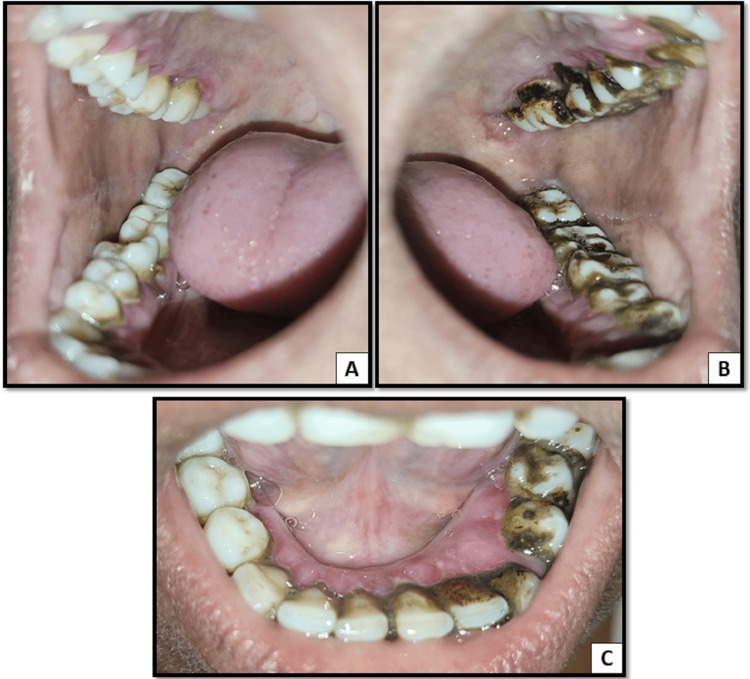
A: Gingival inflammation and enlargement on the palatal aspect on the right side. B: Gingival inflammation and enlargement on the palatal aspect on the left side. C: Gingival inflammation and enlargement on the lingual aspect in the mandibular anterior region.

On careful intra-oral examination, the presence of local factors like plaque and calculus was estimated. There was generalized diffuse gingival enlargement in both the upper and lower jaws. The gingival tissue appeared soggy and was fibrotic, presenting with inflammatory components. Clinically, on a close evaluation, there was a loss of normal gingival contour, loss of stippling, and change in the color and texture of the gingiva. Diffuse erythema and swelling were present on the marginal gingiva with a smooth shiny surface (Figures 1, 2). There was generalized bleeding on probing present in both jaws. On manipulation, there was pus discharge from the enlarged gingiva in the maxillary posterior region on the left side. The probing depth was found to be more than 5 mm.

**Figure 3:**
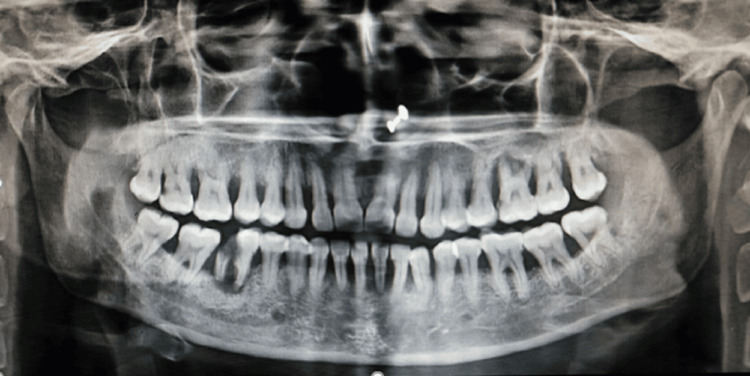
Orthopantomogram showing bone loss in the maxilla and mandible

On the first visit, the patient was given oral hygiene instructions, and an orthopantomogram was done to evaluate the status of the bone in both the maxilla and mandible (Figure 3).

The orthopantomogram depicted a generalized moderate to severe interdental alveolar bone loss, particularly a severe alveolar bone loss in 26 and 46. Proximal caries were seen in 46, and furcation involvement was seen in 16, 26, 36, 37, 46, and 47.

**Figure 4:**
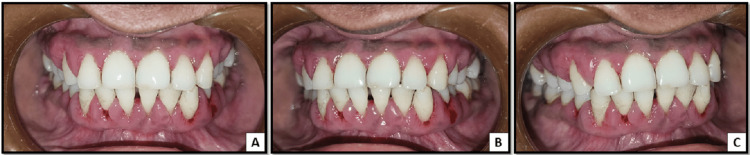
A: Post oral prophylaxis images showing removal of local factors like plaque and calculus in the anterior region. B: Post oral prophylaxis images showing removal of local factors like plaque and calculus in the posterior region on the left side. C: Post oral prophylaxis images showing removal of local factors like plaque and calculus in the posterior region on the right side.

The patient was kept on antimicrobials and analgesic medications for five days. She was prescribed amoxicillin plus clavulanic acid combination 625 mg twice daily after meal for five days, diclofenac and paracetamol combination 100 mg twice daily after meal for five days, Fibrogard tablet twice daily after meal for five days, and pantoprazole once daily before meal for five days, and then she was referred to the Department of Periodontics for oral prophylaxis and subgingival curettage. Figure 4 shows the immediate post-oral prophylaxis intra-oral status.

After scaling and root paning, she was prescribed chlorhexidine mouthwash for 10 days and was recalled for a root planning procedure in the Department of Periodontics.

**Figure 5:**
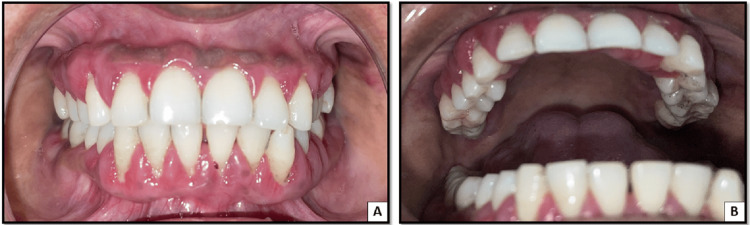
A: Follow-up visit after seven days showing reduced gingival inflammation and gingival enlargement in the anterior region. B: Follow-up visit after seven days showing reduced gingival inflammation and gingival enlargement in the posterior region.

On the follow-up visit after seven days, there was a complete resolution of gingival enlargement. Inflammation and erythema were reduced (Figure 5). Bleeding on probing was absent, and the generalized probing depth was reduced to 3 mm.

She was advised regular follow-ups at every 3-month interval.

## Discussion

As the oral mucosa is continuously exposed to both internal and external stimuli, it can develop a wide spectrum of disorders, from inflammatory to neoplastic, developmental to reactive [4].

Humanity has been aware of the clinical signs of gingival disorders for about four millennia. Our understanding of the nature of gingival illnesses did not develop until the latter part of the 20th century. There is a growing consensus that gingivitis is actually a spectrum of diseases that result from a number of distinct processes rather than a single disease While there are many different ways to cause inflammation in the gingival tissues, including trauma, chemicals, extreme temperatures, ionizing radiation, viruses, fungi, immune defects, and so on, gingival pathologies are currently limited to gingival tissues and caused by dental plaques [5].

Every alteration in the gingival tissues manifests as some level of irritation. Accurately identifying the etiology is important for timely management. However, among the plethora of conditions that could be categorized by causative factors and pathological alterations, by criteria that include the extent of disease and where it is exactly located, or by the grade of enlargement, the skills of clinicians are tested when reaching an accurate diagnosis [1].

Upon observing a patient with gingival enlargement, a thorough visual examination is performed to identify any anomalies in the gingival contours, texture, and color. These findings are then compared to the normal norms. To rule out probable systemic variables and illnesses, a thorough medical history is obtained in addition to a visual inspection. A precise evaluation is essential for organizing the therapy approach and follow-up stage [1].

Sandhu et al. described the case of a young, otherwise healthy, male patient who had long-standing gingival development affecting the front portions of both the upper and lower arches. Through the use of a surgical procedure known as gingivectomy, the overgrowth was eliminated and a great aesthetic outcome was obtained. Following a 15-day period of observation, the healing process was deemed adequate, and no adverse effects were observed [6].

Tomar et al. depicted the case of a 20-year-old female patient whose main complaint was swelling in the lower front area of her teeth's gums. The patient found it challenging to practice proper oral hygiene because of the soft, friable, and spontaneously bleeding enlargement. Surgery was performed in order to get a satisfactory cosmetic result [7].

Singh et al. in a case report presented a female patient, age 18, who presented to the Mithila Minority Dental College and Hospital's Department of Periodontology in Bihar, India. The patient said that for six months, the upper gums in front of the tooth area had swollen. She also reported experiencing bleeding when brushing her gums. For improved function and appearance, a surgical operation and periodontal therapy were scheduled as a part of the treatment plan [8].

Ozgoz et al. presented a case of a 35-year-old female patient who complained of bleeding and gingival growth when she visited the periodontology department at Atatürk University. The gingival hypertrophy between the top left and right first incisors was discovered during a clinical examination. When probed, the swollen gingiva was edematous and prone to bleeding. First, extensive scaling, root planing, and dental plaque control techniques were used [9].

Understanding the underlying pathology and cause of gingival enlargement is the foundation for treating the condition. Each type of enlargement has a different course of treatment depending on the signs and symptoms evaluated clinically. Surgical therapy should not begin until phase I therapy has been implemented. The course of treatment differs depending on the kind of enlargement. It is a common practice to combine surgical and nonsurgical therapies, depending on the patient’s requirements. It is also essential to consider aesthetic and functional requirements [10].

A complete debridement of deposits along with extensive scaling and root planing comprise the therapy. Tissue shrinks as a result, albeit not completely [11].

Prolonged gingival expansion may also exhibit fibrotic elements; hence, total tissue shrinkage does not occur in these circumstances. Surgical therapy should be taken into consideration if the gingival tissue does not return to its normal state after phase I therapy has been implemented. The surgical treatment entails flap operations or gingivectomy procedures. These dealings are recommended when the gingival tissue remains soft and edematous. The chosen course of treatment in cases when the tissues are fibrotic and firm is flap surgery. It is necessary to remove extra gingival tissues while also taking into account the preservation of the keratinized, connected gingiva. A surgical blade is used to cut the tissue away from the mucosa at its base during the process. In order to guarantee deposit exposure and create a scalloped gingival contour, the gingiva located in the interdental area is incorporated within the incision if the lesion extends inter-proximally. The surfaces of roots are scaled and planed, and a thorough irrigation of the area is done following the full removal of the swollen tissue and appropriate accessibility. After one week, the periodontal dressing is taken off. The appointment after the surgical procedure could be planned within two weeks, depending on the scope of the surgery, to allow for more healing. With a gingivectomy, the secondary aim facilitates healing. Healing after a flap operation occurs primarily through intention. Following the debridement of the root surface and the elevation of the mucoperiosteal flap, sutures are placed [12].

Thus, the primary takeaway message from this case is to emphasize on the detailed case history of the patient in order to rule out all the possible causes followed by a careful clinical examination, which will lead to the final diagnosis, Once diagnosed, early interventions should be done to limit the progress and to relieve the symptoms [13].

## Conclusions

Gingival enlargement refers to the increased size of gingival tissues due to multifactorial etiologies. A thorough medical history and family history are important for the accurate diagnosis of this condition. Local factors, such as plaque and calculus, tend to be the most common underlying factors for gingival enlargement and inflammation. Recognizing the etiologic factor and prompt intervention can limit the progression of the condition and bone loss can also be halted. Strict instructions related to oral hygiene and extensive oral prophylaxis comprise the initial treatment protocol. The surgical approach can be taken into consideration if the case becomes refractory to the initial phase I treatment protocol. 
